# *DC-SIGN* (CD209) Promoter −336 A/G (rs4804803) Polymorphism Associated with Susceptibility of Kawasaki Disease

**DOI:** 10.1100/2012/634835

**Published:** 2012-05-02

**Authors:** Hong-Ren Yu, Wei-Pin Chang, Lin Wang, Ying-Jui Lin, Chi-Di Liang, Kuender D. Yang, Chiu-Ming Kuo, Yi-Chuan Huang, Wei-Chiao Chang, Ho-Chang Kuo

**Affiliations:** ^1^Department of Pediatrics, Kaohsiung Chang Gung Memorial Hospital, Kaohsiung 83301, Taiwan; ^2^College of Medicine, Chang Gung University, Kaohsiung, Taiwan; ^3^Department of Healthcare Management, Yuanpei University, Hsinchu 30015, Taiwan; ^4^Department of Medical Research and Pediatrics, Show Chwan Memorial Hospital in Chang Bing, Changhua 505, Taiwan; ^5^Department of Nursing, Chang Gung Memorial Hospital, Chiayi, Taiwan; ^6^Department of Medical Genetics, College of Medicine, Kaohsiung Medical University, Kaohsiung 80708, Taiwan; ^7^Cancer Center, Kaohsiung Medical University Chung-Ho Memorial Hospital, Kaohsiung 807, Taiwan

## Abstract

Kawasaki disease (KD) is characterized by systemic vasculitis of unknown etiology. High-dose intravenous immunoglobulin (IVIG) is the most effective therapy for KD to reduce the prevalence of coronary artery lesion (CAL) formation. Recently, the *α*2, 6 sialylated IgG was reported to interact with a lectin receptor, specific intracellular adhesion molecule-3 grabbing nonintegrin homolog-related 1 (SIGN-R1) in mice and dendritic cell-specific intercellular adhesion molecule-3 grabbing nonintegrin (*DC-SIGN*) in human, and to trigger an anti-inflammatory cascade. This study was conducted to investigate whether the polymorphism of *DC-SIGN* (CD209) promoter −336 A/G (rs4804803) is responsible for susceptibility and CAL formation in KD patients using Custom TaqMan SNP Genotyping Assays. A total of 521 subjects (278 KD patients and 243 controls) were investigated to identify an SNP of rs4804803, and they were studied and showed a significant association between the genotypes and allele frequency of rs4804803 in control subjects and KD patients (*P* = 0.004 under the dominant model). However, the promoter variant of *DC-SIGN* gene was not associated with the occurrence of IVIG resistance, CAL formation in KD. The G allele of *DC-SIGN* promoter −336 (rs4804803) is a risk allele in the development of KD.

## 1. Introduction

Kawasaki disease (KD), mucocutaneous lymphnode syndrome, is a systemic vasculitis that predominantly affects children under the age of five years. Although the cause is still unknown, KD is the most common cause of acquired heart disease during childhood in the developed countries at this time. Coronary artery lesions (CAL) are the major complications of KD. There is a 15–25% chance of CAL developing in KD patients without early treatment [1]. Although the exact therapeutic mechanisms have not been fully established, high-dose intravenous immunoglobulin (IVIG) is the most effective therapy for KD to reduce the prevalence of CAL [2].

Many potential mechanisms of action for IVIG have been suggested [3]. Of them, at least three main mechanisms are suggested to explain the anti-inflammatory function of high dose IVIG: first, high-dose IgG saturates the neonatal FcRs (FcRn) and leads to the increased catabolism of autoantibodies; second, high-dose IgG saturates the activating Fc*γ* receptors (Fc*γ*Rs) and prevents autoantibody-mediated activation of leukocytes; third, high-dose IgG increases the cell surface expression of inhibitory Fc*γ* receptors [4]. A single, N-linked glycosylation site exists at the amino acid 297 in the heavy chain of all IgG subclasses with approximately 10% terminating in sialic acid [5]. Recently, the *α*2, 6 sialylated IgG was reported to interact with a lectin receptor, specific intracellular adhesion molecule-3 grabbing nonintegrin homolog-related 1 (SIGN-R1) in mice and dendritic cell-specific intercellular adhesion molecule-3 grabbing non-integrin (*DC-SIGN*) in humans, and to trigger an anti-inflammatory cascade that promotes the upregulation of inhibitory Fc*γ*Rs on inflammatory macrophages [6].

There has been some evidence demonstrating the role of *DC-SIGN *promoter variants in the susceptibility to or the protection against various infectious diseases, such as dengue fever, tuberculosis, and AIDS [7–9]. However, whether *DC-SIGN *promoter variants have effects on susceptibility to KD is still unknown. Since *DC-SIGN*, also known as CD209, is so important for the anti-inflammatory functions of IVIG, it is reasonable to hypothesize that a functional single nucleotide polymorphism (SNP) in the CD209 molecule will be involved in the pathogenesis of KD or response to IVIG treatment. We hypothesized that the SNP rs4804803 of *DC-SIGN* promoter may be involved in the susceptibility to KD, CAL formation, coronary artery fistula formation, and IVIG treatment response in KD patients. To test this hypothesis, we conducted a case-control study involving 278 patients with KD and 243 controls.

## 2. Materials and Methods

### 2.1. Patients Studied

All patients studied were children who fulfilled the diagnostic criteria for KD and were admitted for IVIG treatment at Chang Gung Memorial Hospital—Kaohsiung Medical Center, from 2002 and 2009. All patients were treated with a single high-dose IVIG (2 g/kg) over a 12-hour period [10–12]. This study was approved by the Institutional Review Board of Chang Gung Memorial Hospital with written consent statement. We excluded patients who did not fit the diagnostic criteria of KD. CAL was defined by the internal diameter of the coronary artery being greater than 3 mm (4 mm, if the subject was over the age of 5 years) or the internal diameter of a segment being at least 1.5 times that of an adjacent segment, as observed in the echocardiogram [13, 14]. KD patients with coronary artery ectasia or dilatation which was disappearing within the initial 6–8 weeks after the onset of illness was defined as transient CAL [3]. The diagnosis of coronary artery fistula (CAF) was made mainly by pulsed Doppler and color flow imaging [15]. IVIG treatment responsiveness was defined as defevrscence 48 hrs after the completion of IVIG treatment and no fever (temperature, >38°C) recurrence for at least 7 days after the initial IVIG treatment with marked improvement of inflammatory signs [16]. Patients with IVIG resistance received another dose of IVIG (1-2 g/kg) or other anti-inflammatory regiments. Children who were admitted for upper and/or lower respiratory tract infections (including acute bronchiolitis, acute pharyngitis, acute bronchitis, croup, and acute tonsillitis) were also collected as control subjects for comparison during the same study period, as we have previously described [12].

### 2.2. Genotyping of CD209 rs4804803 SNP

Genomic DNA was isolated from heparin-anticoagulated blood samples using a standard phenol-chloroform extraction followed by 70% alcohol precipitation. Genotyping for the CD209 variant (−336 A/G; rs4804803) was carried out using Custom TaqMan SNP Genotyping Assays (Applied Biosystems, Foster City, CA, USA). The primer sequences were 5′-GGACAGTGCTTCCAGGAACT-3′ (forward) and 5′-TGTGTTACACCCCCTCCACTAG-3′ (reverse). The TaqMan minor groove binder probe sequences were 5′-TACCTGCCTACCCTTG-3′ and 5′-CTGCCCACCCTTG-3′. The probes were labeled with the TaqMan fluorescent dyes VIC and FAM, respectively. The PCR was conducted in total volume of 15 *μ*L using the following amplification protocol: denaturation at 95°C for 10 min, followed by 40 cycles of denaturation at 94°C for 20 s, followed by annealing and extension at 60°C for one minute. After the PCR, the genotype of each sample was determined by measuring the allele-specific fluorescence in the ABI Prism 7500 Sequence Detection System, using SDS 1.1 software for allele discrimination (both Applied Biosystems). To validate the genotyping by real-time PCR analysis, 100 PCR products were subject to restriction fragment length polymorphism (RFLP) analysis with MscI restriction enzyme (New England Biolabs, Beverly, MA, USA) and showed a 100% identical result between these two genotyping systems as noted in our previous report [17].

### 2.3. Statistics Analyses

The Hardy-Weinberg equilibrium was first checked. The statistical differences between case and control in genotype and allele frequency were assessed by chi-square test. The statistical differences in the genotype and allele frequency of KD patients with and those without CAL formation, aneurysm formation, patients responding to IVIG, and those showing resistance were assessed using chi-square test. SAS 9.1 for Windows was used for data analysis.

## 3. Results

### 3.1. *DC-SIGN* −336 (rs4804803) A/G Polymorphism Was Associated with the Susceptibility of Kawasaki Disease

In this study, a total of 278 KD patients were included, of which 35 patients (12.6%) were resistant to initial IVIG treatment, 42 patients (15.1%) had CAL formation and 13 patients (4.7%) developed coronary artery fistula. In this study, as shown in Table 1, the difference of rs4804803 genotype between KD patients and controls was statistically significant (*P* = 0.004, dominant model, Table 1). Minor G allele of rs4804803 was over represented in the KD patients as compared with the controls (8.1 versus 3.5%).

### 3.2. No Significant Association of *DC-SIGN* −336 (rs4804803) A/G Polymorphism with IVIG Treatment Response and CAL Formation in KD Patients

We further evaluate the relationship between rs4804803 and the risk of IVIG resistance or CAL formation. As shown in the Tables 2 and 3, the frequency of AA genotype was higher in the patients with IVIG responsiveness (86.0 versus 77.1%) and without CAL formation (85.2 versus 83.3%). The genotype or allele frequency of rs4804803, however, was not statistically associated with IVIG resistance (Table 2) or CAL formation (Table 3).

### 3.3. No Significant Association of *DC-SIGN* −336 (rs4804803) A/G Polymorphism with Coronary Artery Fistula Formation in KD Patients

To further identify the role of rs4804803 of CD209 in the pathogenesis of coronary artery fistula in KD patients, we performed a subset analysis in cases that were reported as having fistula formation (13/278, 4.7%). Subset analysis between cases with coronary artery fistula and rs4804803 did not yield any significant results (Table 4).

## 4. Discussion

*DC-SIGN* is a transmembrane lectin receptor on dendritic cells with multiple immune modulation function [18] *DC-SIGN* can recognize many pathogens, such as viruses (HIV-1, dengue, and measles virus) [19–21], bacteria (*Helicobacter pylori*, *Mycobacterium tuberculosis*) [22], and fungi (*Candida albicans* and *Aspergillus fumigatus*) [23] contributing to generation of pathogen-tailored immune responses and immunosuppressive response by the MAPK pathway in DCs [24]. The real cause of KD remains unknown. It is generally accepted that KD results from an undefined infectious process trigger in a genetically predisposed individual [25]. A genetic predisposition is suggested based on clinical and epidemiologic features [1, 26]. In this study, we investigated whether the polymorphism of *DC-SIGN* (CD209) promoter −336 A/G (rs4804803) was associated with susceptibility and CAL formation in KD. Our study showed that the allele −336G was associated with susceptibility to KD. To the best of our knowledge, this is the first study to explore the association between *DC-SIGN* polymorphisms and susceptibility to KD.

Immunoglobulin is well known for its defensive role in pyogenic infection. In addition to its protective role, immunoglobulin G (IgG) was also noted to have anti-inflammatory effects at high doses. Recently, the *α*2, 6 sialylated IgG was reported to interact with a lectin receptor, SIGN-R1 in mice and *DC-SIGN* in humans, and to trigger an anti-inflammatory cascade. Thus it is reasonable to hypothesize that a functional SNP in the *DC-SIGN* molecule will be involved in the response to IVIG treatment. However, in our study, we found the variant and haplotype of −336A/G in the *DC-SIGN* gene did not associate with the occurrence of IVIG resistance or CAL formation in KD. Because of its highly polymorphic nature and numerous SNPs of *DC-SIGN* gene [27–29], further investigation into other candidate SNPs contributing to KD morbidity is needed. Besides dendritic cells, IVIG was observed to affect many other cells, including endothelial cells, monocytes, neutrophils, and T and B cells [30–32]. The numerous effects of IVIG therapy also partly explain there not being an association of −336A/G SNP of the *DC-SIGN* gene with IVIG resistance in KD.

There were some limitations with regards to this study. First, the relatively small sample size of this study might prevent some of the detected associations from being statistically significant. Second, our study results need to be validated across different populations. Since the incidence of KD in Asian populations is much greater than among Caucasians [1], the host's genetic background must be considered in the study of KD. In our control group, the frequency of the −336G *DC-SIGN* gene allele was 3.5%. This result agreed with previous reports showing very low −336G *DC-SIGN* gene allelic frequency in Asians [33, 34]. The highest −336G allelic frequency was found in African populations (35–48%), next in Caucasian populations (20%), and the lowest was observed in Asians [8, 33].

In conclusion, from our study, we found the G allele of *DC-SIGN* promoter −336 (rs4804803) to be a risk allele in the development of KD in a Chinese population. Further studies to explore the effects of other SNPs of *DC-SIGN* or a combination of genes are needed.

## Figures and Tables

**Table 1:** Genotype frequencies for CD209 −336 A/G and Kawasaki disease susceptibility.

	Genotype	Case (%) (*n* = 278)	Control subjects (%) (*n* = 243)	Allele	Case (%) (*n* = 278)	Control subjects (%) (*n* = 243)	Genotype *P* value	Dominant *P* value	Recessive *P* value	Allelic *P* value
CD209 −336 A/G	GG	3 (1.1)	0 (0.0)	G	45 (8.1)	17 (3.5)	0.008	0.004	0.104	0.002
	GA	39 (14.0)	17 (7.0)	A	511 (91.9)	469 (96.5)				
	AA	236 (84.9)	226 (93.0)							

**Table 2:** Genotyping and allele frequency of CD209 −336 A/G in patients resistant and responsive to intravenous immunoglobulin (IVIG) treatment.

	Genotype	Resistant (%) (*n* = 35)	Responsive (%) (*n* = 243)	Allele	Resistant (%) (*n* = 35)	Responsive (%) (*n* = 243)	Genotype *P* value	Dominant *P* value	Recessive *P* value	Allelic *P* value
CD209 −336 A/G	GG	1 (2.9)	2 (0.8)	G	9 (12.9)	36 (7.4)	0.290	0.171	0.276	0.118
	GA	7 (20.0)	32 (13.2)	A	61 (87.1)	450 (92.6)				
	AA	27 (77.1)	209 (86.0)							

**Table 3:** Genotyping and allele frequency of CD209 −336 A/G in patients with coronary artery lesion (CAL) and without CAL.

	Genotype	CAL (%) (*n* = 42)	Without (%) (*n* = 236)	Allele	CAL (%) (*n* = 42)	Without (%) (*n* = 233)	Genotype *P* value	Dominant *P* value	Recessive *P* value	Allelic *P* value
CD209 −336 A/G	GG	0 (0.0)	3 (1.3)	G	7 (8.3)	38 (8.1)	0.673	0.760	0.463	0.930
	GA	7 (16.7)	32 (13.5)	A	77 (91.7)	434 (91.9)				
	AA	35 (83.3)	201 (85.2)							

**Table 4:** Genotyping and allele frequency of CD209 −336 A/G in patients with fistula or without fistula.

	Genotype	Fistula (%) (*n* = 13)	Without (%) (*n* = 265)	Allele	Fistula (%) (*n* = 13)	Without (%) (*n* = 265)	Genotype *P* value	Dominant *P* value	Recessive *P* value	Allelic *P* value
CD209 −336 A/G	GG	0 (0.0)	3 (1.1)	G	2 (9.4)	43 (8.1)	0.921	0.977	0.700	0.939
	GA	2 (15.4)	37 (14.0)	A	24 (90.6)	487 (91.9)				
	AA	11 (84.6)	225 (84.9)							
